# *Drosophila* rely on learning while foraging under semi-natural conditions

**DOI:** 10.1002/ece3.783

**Published:** 2013-09-23

**Authors:** Vukašin Zrelec, Marco Zini, Sandra Guarino, Julien Mermoud, Joël Oppliger, Annabelle Valtat, Valérian Zeender, Tadeusz J Kawecki

**Affiliations:** 1 Department of Ecology and Evolution, University of LausanneLausanne, Switzerland; 2 School of Biology, University of LausanneLausanne, Switzerland

**Keywords:** Cognitive ecology, *Drosophila melanogaster*, foraging, insects, learning, resource preference

## Abstract

Learning is predicted to affect manifold ecological and evolutionary processes, but the extent to which animals rely on learning in nature remains poorly known, especially for short-lived non-social invertebrates. This is in particular the case for *Drosophila*, a favourite laboratory system to study molecular mechanisms of learning. Here we tested whether *Drosophila melanogaster* use learned information to choose food while free-flying in a large greenhouse emulating the natural environment. In a series of experiments flies were first given an opportunity to learn which of two food odours was associated with good versus unpalatable taste; subsequently, their preference for the two odours was assessed with olfactory traps set up in the greenhouse. Flies that had experienced palatable apple-flavoured food and unpalatable orange-flavoured food were more likely to be attracted to the odour of apple than flies with the opposite experience. This was true both when the flies first learned in the laboratory and were then released and recaptured in the greenhouse, and when the learning occurred under free-flying conditions in the greenhouse. Furthermore, flies retained the memory of their experience while exploring the greenhouse overnight in the absence of focal odours, pointing to the involvement of consolidated memory. These results support the notion that even small, short lived insects which are not central-place foragers make use of learned cues in their natural environments.

## Introduction

Learning can be defined as a change in behaviour driven by the memory of previous experience (Davis 2005) and may help an animal to adapt its behaviour in response to changing environmental circumstances (Dukas 2004). While many vertebrates have been shown to rely on learning under natural conditions (Dukas 2004), evidence for ecological relevance of learning in insects is mostly limited to bees (e.g., Menzel and Muller 1996; Hill et al. 1997; Menzel et al. 2005; Raine and Chittka 2008; Ings et al. 2012), parasitoids (e.g., van Nouhuys and Kaartinen 2008; Hoedjes et al. 2011; Froissart et al. 2012; Thiel et al. 2013) and macrolepidoptera (e.g., Rausher 1978; Stanton 1984; Cunningham et al. 2004; Snell-Rood and Papaj 2009). Several of those studies provide ecological underpinning for specific laboratory assays of learning in those insect groups (e.g., Menzel and Muller 1996; Raine and Chittka 2008; Hoedjes et al. 2011; Thiel et al. 2013). However, not all laboratory learning assays extrapolate to nature. For example, although honey bees remember flowers associated with perceived danger in the laboratory, they are apparently unable to learn to avoid flowers with predatory crab spiders in the field (Dukas et al. 2005). Thus, even though many more insect species have been shown to learn in laboratory conditioning assays, extrapolating from such assays to nature may be problematic, especially when the assays do not have an obvious connection with the animal's ecology.

**Figure 1:**
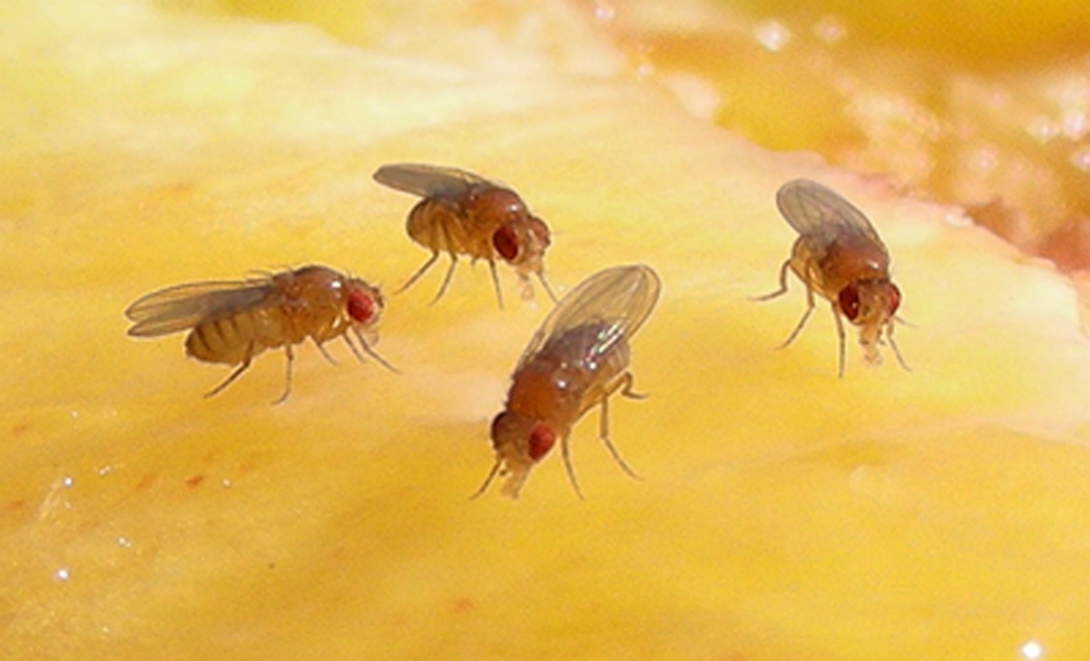
Fruit flies *Drosophila melanogaster* feeding on decomposing fruit. Photo copyright T. J. Kawecki.

A case in point is the fruit fly *Drosophila melanogaster*, a favourite model species for studying the genetics, neural mechanisms and evolution of associative learning (Fig. 1; Davis 2005; Kawecki 2010). Several experimental paradigms for quantifying associative learning in *Drosophila* in the laboratory have been developed, involving associations of odours or visual cues with shock, heat, bitter taste or sugar reward (e.g., Tempel et al. 1983; Tully and Quinn 1985; Scherer et al. 2003; Foucaud et al. 2010). The use of these paradigms has greatly advanced our understanding of the mechanisms of learning, but their relevance to what *Drosophila* may learn in nature is unclear. In these assays the flies are either immobilized or confined to a very small space; the experimental stimuli are strong and the flies cannot avoid perceiving them. A few somewhat more ecologically relevant laboratory assays have demonstrated that experience can change flies' mating and oviposition behaviour (Wolf and Heisenberg 1991; Mery and Kawecki 2002; Dukas 2005b). Still, even those assays confine flies to highly spatially restricted and very simple environments. It is not clear to what extent those learned responses would scale up to natural environments, where the spatial scale is orders of magnitude larger and a multitude of stimuli compete for the fly's attention. For example, a male fruit fly constrained with an unreceptive female in a small space subsequently refrains from courting even receptive females for several hours (“courtship conditioning”; Siegel and Hall 1979); however, this does not occur under less constrained conditions more akin to flies' natural environment (Dukas 2005a). Similarly poor correspondence between laboratory and field have been found for an innate behavioural pattern – circadian activity rhythm (Vanin et al. 2012).

Not having a nest or brood care, fruit flies may not need the cognitive abilities required for homing, while their short lifespan under natural conditions (Rosewell and Shorrocks 1987) would limit their chances to benefit from past experience. It could be argued that the learning observed in the laboratory indirectly supports the relevance of learning ability to fitness in nature: if learning were not beneficial, this costly trait (Mery and Kawecki 2003) would be eliminated by natural selection. Yet, it is also possible that some basic level of learning ability is conserved as a by-product of general neuronal plasticity (important in nervous system development), even if learning is ecologically irrelevant or costly (Dukas 2009). So the fact that fruit flies learn in the laboratory does not necessarily imply that learning is ecologically relevant for this animal in nature.

Knowing if and what fruit flies learn in nature would throw light on the evolutionary forces maintaining learning ability and provide ecological underpinning for the neuroscience-oriented research on learning in this model species. Furthermore, learning has been proposed to buffer populations against environmental fluctuations (Stephens 1991), affect predator-prey population dynamics (Ishii and Shimada 2012), facilitate expansion into novel habitats (Sutter and Kawecki 2009), modulate evolutionary change (Paenke et al. 2007) and initiate speciation (Thorpe and Jones 1937; Dukas 2005b). Thus, showing that even a small, short lived, non-social insect makes use of learning in nature would greatly extend the potential taxonomic relevance of those hypotheses.

A few studies have addressed the effect of experience on behaviour of *Drosophila* under field conditions, with mixed results. Jaenike (1985, 1986)) reports increased attraction of *D. melanogaster* (and *Drosophila tripunctata*) in field releases to food on which the flies had been previously kept in the laboratory for a week. Similarly, *D. melanogaster* which emerged from pupa in immediate vicinity of a particularly flavoured food were subsequently more attracted to that flavour under field conditions (Jaenike 1988). However, other studies with similar design (Hoffmann and Turelli 1985; Hoffmann 1988; Turelli and Hoffmann 1988), found no or inconsistent evidence for effects of prior food exposure on its subsequent attractiveness to flies released in the field. All those studies involved non-differential learning; i.e., the flies only acquired experience of one food type rather than comparing food sources of different palatability. Thus, where effects were detected, they could reflect imprinting or sensitization (or habituation in cases where reduced attraction to previously experienced food was found; Hoffmann and Turelli 1985; Turelli and Hoffmann 1988), irrespective of food quality. Furthermore, while flies possess consolidated memory and can remember an association between shock and odour for over at least 24 h in laboratory assays (Dubnau and Tully 1998; Isabel et al. 2004; Mery et al. 2007), it is not clear if consolidated memory is relevant to food choice in nature.

In this paper we address the ecological relevance of learning in fruit flies by testing if they learn about food quality, retain this information overnight, and use it to choose between food types in a greenhouse setting. This setting emulates the natural environment in that expanse of space the flies can explore is vast compared to typical laboratory learning assays, and the food sources must be located from afar and approached in flight. Furthermore, plants, pots, soil and greenhouse construction elements provide olfactory and visual complexity and heterogeneity. In our assays, flies were first given the opportunity to learn which of two food substrates (apple- or orange-flavoured) tastes good and which one is less palatable as a result of being laced with quinine (“learning phase”). Subsequently, their attraction to the two food sources was assayed in the greenhouse by setting out traps baited with apple and orange (“test phase”). If their food choices were affected by past experience, the flies should shift their food preference in the test phase towards the previously palatable flavour. We report three experiments which show that fruit flies modify their food preference under the greenhouse conditions based on what they previously learned in the laboratory (experiment 1), that they do so even after remaining in the greenhouse overnight, indicating involvement of consolidated memory (experiment 2), and that they can acquire new learned information while free-flying in the greenhouse (experiment 3).

## Materials and Methods

### General methods

**Figure 2:**
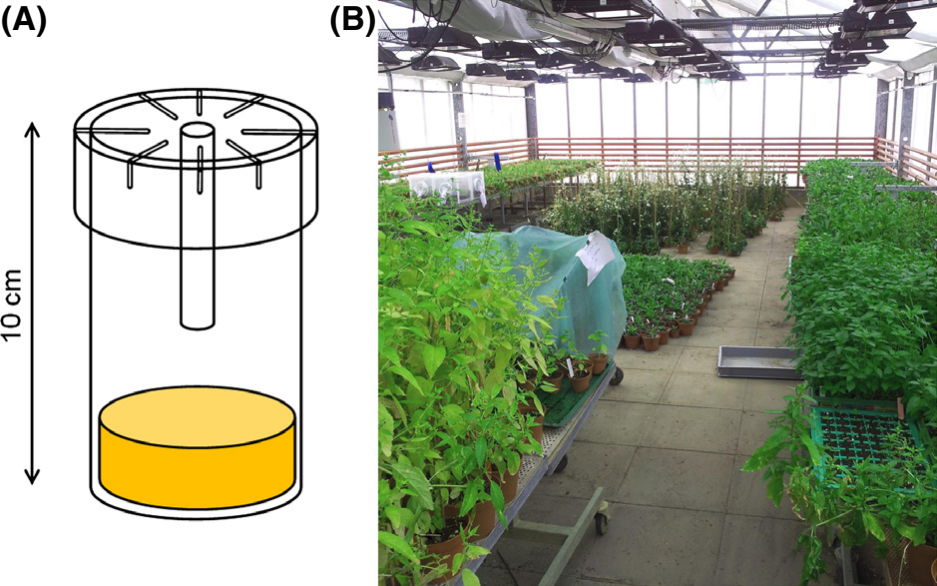
(A) Design of the fly trap used in this study. Clear polystyrene culture vial containing fruit juice jelly sprinkled with dry yeast is capped by a perforated circular lid. Flies are drawn to trap's top by the odour of food emanating through the narrow radial slits (0.6 mm); they cannot pass through the slits and thus converge to the central opening (6 mm diameter) and descend the vertical tube leading to the food. Once inside, the flies tend to cluster on the inner walls of the trap and cannot readily escape. (B) View from the entrance of one of the 18 × 6 m greenhouses where the experiments were performed.

The learning assays were derived from a laboratory oviposition learning paradigm developed by Mery and Kawecki (2002). The food substrates used in the learning assays (both during the learning phase and to bait the traps during the test phase) were made by cooking either orange or apple juice with 22 g/L agar. To make a substrate unpalatable (bitter), quinine hydrochloride was added at the concentration of 7 g/L. Substrates were poured into Petri dishes (for the learning phase) and into the traps (for the test phase), and allowed to cool before a pinch of dry yeast was added onto their surface. The traps were assembled from 160 mL polystyrene vials covered with 45 mm diameter plastic lids perforated with several radial slits with a small central opening leading into a narrow vertical descending tube (Fig. 2A). Flies could thus readily detect and be drawn to odours emanating from the substrate within each trap but once inside could not escape.

We used three large (6 × 14 m) greenhouses (Fig. 2B); however, only one greenhouse was available at any particular time for reasons beyond our control. The greenhouses were illuminated by natural ambient light; additionally halogen lamps set to 12:12 h light–dark cycle were activated when the natural light was low. Temperature varied between 18 and 25°C, relative humidity between 30% and 65%. Numerous potted plants of various species were distributed in the greenhouses. Plants were densely packed on tables which covered most of the greenhouses' surface; the species represented were not under our control and varied over time. To assay flies' choice between orange and apple odours eight pairs of traps, consisting of one orange- and one apple-baited about 5 cm distant from each other, were distributed around the perimeter of the greenhouse. The flies were released at the centre of the greenhouse, at least 2 m from the nearest trap pair. The released flies were observed roosting on the plants and potted soil in the greenhouses, which allowed them to obtain moisture, but no apparent food sources were available (except for the substrates provided in experiment 3; see below).

We used an outbred *D. melanogaster* population derived from a natural population in Valais, Switzerland in 2007. Flies were reared at 25°C and 60% relative humidity under a 12: 12 light: dark cycle on standard cornmeal-sugar-yeast medium. The experiments were done with flies aged 3–10 days, sexes mixed. Flies used in the experiments were counted under light CO_2_ anaesthesia, marked with red or yellow fluorescent powder according to the learning treatment (see below) and kept for 12 h on an agar substrate before the start of the experiments. The marking was effective as all of the trapped flies invariably showed traces of the powder; this also indicates that no flies entered the greenhouse from outside. The experiments described below were carried out over the span of a year, with at least 3 day interval between successive experiments in the same greenhouse. In a pilot study we verified that no flies were recaptured after 3 days in the greenhouse with no food provided, consistent with the known starvation time in *Drosophila* (Rion and Kawecki 2007). Thus, flies released in one experimental run could not “contaminate” the following run.

Following the majority of choice-based assays of olfactory associative learning in *Drosophila* (e.g., Davis 2005; Mery and Kawecki 2005; Mery et al. 2007; Placais and Preat 2013), the hypothesis of learning was tested by comparing the relative attraction to the two odours between flies that experienced opposite association between odours and the unconditioned stimulus (here food quality). That is, we tested if flies previously exposed to palatable apple substrate and quinine-laced orange substrate were more likely to be recaptured in the apple traps than flies subject to the opposite treatment.

### Experiment 1: Laboratory learning, immediate greenhouse testing

This experiment aimed to test if flies modify their choice of food odour in response to experience acquired in the laboratory immediately before. For the learning treatments, groups of 200 flies marked with red or yellow fluorescent powder were transferred to 58 × 80 × 97 mm polystyrene boxes with one 35 mm Petri dish with orange and one with apple substrate. In one treatment orange was supplemented with quinine (i.e., flies learned that apple was more palatable); in the other treatment apple contained quinine (i.e., flies learned that orange was more palatable). Flies laid eggs on both substrates during this phase (although as expected fewer on the one laced with quinine), indicating that both substrates were sampled. After 15 h of this learning experience all flies were released in the greenhouse where the traps baited with apple and orange were already set up. The traps were collected 10 h after fly release. This experiment was performed twice in October 2011 in greenhouse #1, with 400 flies per treatment released in each experimental run.

Flies in each trap were counted according to the trap flavour and learning treatment (recognized by the marking colour). The proportion of flies caught in apple rather than orange traps is a measure of their preference for the odour of the apple substrate. This proportion was expected to be higher for flies which learned during the learning phase that apple was more palatable (i.e., experienced orange with quinine) than for flies which learned that orange was more palatable. To test this hypothesis we compared the proportion of flies recaptured in apple traps between learning treatments with a nominal logistic model (with JMP v. 8; SAS Institute Inc.), using treatment as the effect and trap pair as block. To check for robustness of the results against heterogeneity across traps, we additionally performed the Cochran–Mantel–Haenszel test stratified by trap pair (using JMP v. 8). The results of both analyses were in a very good agreement, and we only report the Cohran–Mantel–Haenszel test because it was in all cases slightly more conservative. The proportion of flies recaptured was also compared between conditioning treatments with nominal logistic regression. For graphs, standard errors of proportions were calculated as (*q*(1 − *q*)/*N*)^1/2^ where *q* is the estimate of proportion and *N* is the sample size.

### Experiment 2: Laboratory learning, overnight retention in the greenhouse

Here, we tested whether memory of experience acquired in the laboratory is retained in the greenhouse overnight in the absence of the food stimuli. Flies were allowed to learn for 8 h in groups of 100 individuals as in experiment 1 and then released in the greenhouse in late afternoon. However, the traps were only set up next morning. This experiment was performed twice, in November 2011 in greenhouse #2, with 300 and 800 flies per treatment, respectively. In run 1 the recapture began 16 h after release and lasted for 24 h; in run 2 it began 13 h after release and for logistic reasons lasted only for 7 h. The results were analysed as in experiment 1. Because each experiment was analysed separately and treated as an independent test of the hypothesis, the differences in details of the design between the two runs do not bias the results.

### Experiment 3: Learning acquisition in the greenhouse

In this experiment we tested if flies can learn about food in the greenhouse. Flies were released in the greenhouse without any prior exposure to orange, apple or quinine; rather, they were given an opportunity to learn while free-flying in the greenhouse. To allow them to learn, we set out Petri dishes (100 mm diameter) with a divider in the middle, one half containing the orange and the other the apple substrate; depending on the treatment one of the substrates was supplemented with quinine. We used a single dish with both flavours rather than putting them in separate dishes to maximize the chances of flies experiencing both substrates. Flies were indeed observed walking across the divider (which was flush with the agar surface and so was not obstacle to the flies). Eight such dishes were distributed throughout the greenhouse; the flies were then released and allowed to explore them and the rest of the environment for 24 h, thus having the opportunity to learn about the quality of orange- and apple-flavoured food. Subsequently, the Petri dishes were removed and traps baited with these two substrates were set out as in experiments 1 and 2; the flies in the traps were counted after 8 h. Because the greenhouse could not be divided, we could not simultaneously condition two groups of flies in opposite directions in a single greenhouse. Rather, in four sequential replicate runs of the experiment the orange substrate contained quinine (i.e., flies learned that apple was more palatable), and in four runs apple contained quinine. The first replicate of each treatment was performed in greenhouse #1 in December 2011, the second replicate in February 2012 in greenhouse #3, and the remaining replicates in September 2012 in greenhouse #2. The treatments alternated between successive runs, with 800–1400 flies released per run. The proportion of flies choosing apple versus orange traps was analysed with a Generalized Mixed Model (using PROC GLIMMIX of SAS v. 9.2; SAS Institute Inc.) with binomial error distribution and a logit link function. Treatment was the fixed factor while experimental run nested within treatment was included as a random factor to account for variation in overall attractiveness of apple versus orange traps among experimental runs. This way the experimental run is treated as the main unit of replication when testing the effect of treatment. We also included greenhouse identity in the model, but it was not significant and was removed.

### Unconditioned preference and control for marking effect

This assay aimed to test if the colour of fluorescent powder used to mark flies affects their preference for apple versus orange or their likelihood of being recaptured. It also provided an estimate of unconditioned preference for the two odours, exhibited by flies which did not experience the association between quinine and flavour. It involved the same procedures as Experiment 1 except that neither substrate contained quinine in the learning phase. Thus, flies marked with both colours were exposed to the same treatment, in which they experienced both substrates as palatable. (For simplicity we refer to these flies as being “unconditioned”, keeping in mind that the exposure to two palatable substrates may have changed their relative preference for them.) The first run of this assay, with 400 flies released per marking colour, was performed a few days after the end of experiment 1 in greenhouse #1. Because we saw a shift towards overall greater attractiveness of apple over orange in later experiments (see below), we repeated this assay after the last replicate of experiment 3 in September 2012 in greenhouse #2, with 600 flies released per marking colour. The data were analysed as those from experiment 1 and 2.

## Results

### Experiment 1

**Figure 3:**
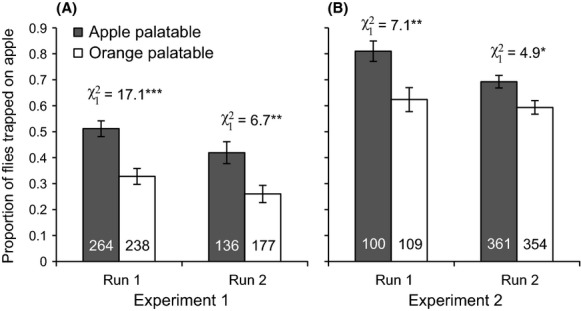
The effect of experience acquired during the learning phase in the laboratory (apple palatable/orange bitter or vice versa) on subsequent odour preference in the greenhouse (the proportion ± SE of flies caught in apple rather than orange traps). (A) Experiment 1: recapture directly after release. (B) Experiment 2: recapture next day (13–16 h) after release. Statistics refer to the Cochran–Mantel–Haenszel test for the effect of treatment; *P < 0.05, **P < 0.01, ***P < 0.001; the numbers above the horizontal axis indicate the total numbers of recaptured flies (i.e., the sample size of the proportion).

In both runs of this experiment flies that previously experienced apple as more palatable were more likely to be found in apple-flavoured traps than flies with the opposite experience (Fig. 3A). Thus, attraction to odours in free-flying flies in the greenhouse environment was modified by their prior learning treatment in the laboratory. The learning treatment affected the recapture probability, but in opposite directions in the two runs: 66% versus 59% in run 1 (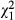 = 3.6, *P* = 0.057), 34% versus 44% in run 2(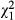 = 8.8, *P* = 0.0093). In both runs the treatment with higher recapture was marked with the yellow powder, suggesting that recapture may be affected by powder colour. The fact that in the two runs the learning treatment had very similar effect on the proportion of flies captured on apple (among total flies captured) confirms that preference for orange versus apple is not biased by recapture rate or marking colour.

### Experiment 2

Despite the long interval (13–16 h) between the release and the recapture, the attraction to food sources in the greenhouse (i.e. the likelihood of being recaptured in orange versus apple traps) was affected by the previous day's experience in the direction consistent with the predictions (Fig. 3B). The proportion of flies recaptured was higher in run 2 than in run 1, possibly reflecting the longer time between release and recapture (16 vs. 13 h); the recapture probability was not affected by the learning treatment (0.33 vs. 0.36 in run 1, 0.45 vs. 0.44 in run 2, both 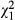 < 1, *P* > 0.4). Irrespective of treatment, flies showed a higher overall preference for apple in Experiment 2 than in Experiment 1.

### Experiment 3

**Figure 4:**
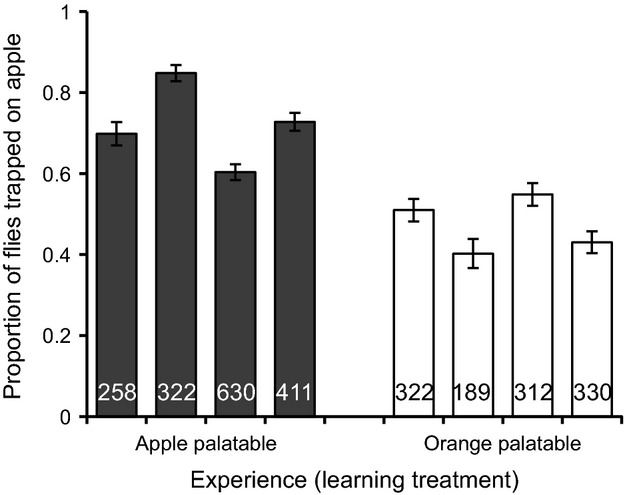
The effect of experience acquired while free-flying in the greenhouse on subsequent food preference (Experiment 3). Each bar shows the proportion (±SE) of flies captured in apple traps in one replicate run of the experiment; the numbers above the horizontal axis indicate the total numbers of recaptured flies (i.e., the sample size of the proportion).

As predicted, flies which had been exposed to palatable apple and bitter orange substrate while free-flying in the greenhouse were subsequently more likely to be found in apple traps than flies with the opposite experience (back-transformed least-square mean proportions ± SE: 0.72 ± 0.04 vs. 0.47 ± 0.05). Despite considerable variation among the replicate runs within treatments (Fig. 4), the difference was significant (*F*_1,6_ = 13.0, *P* = 0.011). The recapture probability tended to be on average higher for flies which experienced palatable apple/bitter orange than for the opposite learning treatment (0.38 ± 0.05 vs. 0.28 ± 0.04), but varied across replicate run so that the overall difference was not significant (*F*_1,6_ = 2.7, *P* = 0.15). Overall, recapture rates in Experiment 3 were somewhat lower than in the other two experiments, presumably reflecting the longer time spent by flies in the greenhouse before recapture and the shorter recapture period.

### Unconditioned preference and control for marking effect

Flies marked with the red powder tended to have somewhat lower recapture probability than those marked yellow (0.59 vs. 0.65; 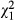 = 3.1, *P* = 0.081, likelihood ratio test). This is consistent with greater recapture of yellow marked flies in experiment 1 (see above). Such a difference in overall recapture probability should not bias our results because they are based on the proportion of flies captured in apple versus orange traps among total flies captured, and because the marking colour was swapped between runs of experiments.

The marking colour did not affect the choice between apple and orange odours: when flies marked with either colour experienced both flavours as palatable (without quinine), similar proportions of them were recaptured in apple-flavoured traps (39% of 235 red marked and 36% of 259 yellow-marked flies recaptured; 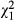 = 0.1, *P* = 0.75, Cochran–Mantel–Haenszel test). This low preference of unconditioned flies for apple traps is consistent with the overall low attractiveness of apple observed in experiment 1 (Fig. 3A), which was carried out a few days before this assay in the same greenhouse. In the second release of unconditioned flies performed after the last run of experiment 3, 67% of 345 flies were recaptured on apple, consistent with the generally higher overall attractiveness of apple observed in the later experiments. The likelihood of being trapped on apple again did not differ between powder colours (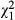 = 0.3, *P* = 0.59).

## Discussion

In this study we demonstrated that the relative attraction of fruit flies flying in a greenhouse environment to alternative odours is affected by the quality of food with which these odours were previously associated. Flies that had experienced palatable apple-flavoured food and unpalatable orange-flavoured food were more likely to be attracted to the odour of apple than flies with the opposite experience. This occurred both when the experience had been acquired under free-flying conditions and under more artificial and confined conditions in the laboratory. The degree to which flies' preference for apple versus orange odour was modified after the flies could learn while free flying in the greenhouse (Experiment 3) was similar to that observed after the flies were first subject to a learning treatment in the laboratory and subsequently tested in the greenhouse (Experiment 1). Furthermore, while the effect of learning in our experiments may seem small, it was similar in magnitude to that reported in a laboratory oviposition learning assay conducted in small boxes preventing flies from flying and only containing the two substrates (Mery and Kawecki 2002). It thus appears that the greenhouse environment, which is richer and more “noisy” in terms of stimuli and which requires flight for exploration, neither poses an obstacle to learning new information, nor significantly impairs flies' ability to make foraging choices based on previously learned information.

The effect of experience on food preference is overlaid on the pre-existing innate preference, and the limited magnitude of this effect means that learning in our study did not lead to reversal of innate preferences. In particular, in experiment 1 flies showed overall greater attraction to the orange than to the apple odour, as confirmed by the release of unconditioned flies. While flies that experienced orange as unpalatable significantly shifted the relative preference away from orange and towards apple, they still chose apple at most half of the time. Conversely, in later experiments (2 and 3) flies were in general considerably more attracted to apple (confirmed by the second release of unconditioned flies), so that even those that experience orange as palatable and apple as bitter tended to choose apple traps as often or more often than orange traps. However, similar observations have been made in laboratory learning assays (Mery and Kawecki 2004). Thus the inability of learning to reverse pre-existing innate biases points to limits on *Drosophila* learning, but it does not negate the fact that learning, defined as a changed of food odour choice based on past experience, occurred in our experiments. Furthermore, if an animal shows a strong but maladaptive innate preference for a particular food, even a small reduction of this preference may have large fitness consequences. In an extreme case when the innately preferred food turns out to be lethally toxic, reducing the likelihood of choosing it from 90% to 80% would double fitness.

While the greenhouse obviously still differs from a natural environment, its spatial scale and relative complexity are presumably more relevant to the natural ecology of *Drosophila* than the confines of laboratory learning assays (see Introduction). A few previous studies (Jaenike 1985, 1986, 1988) demonstrated an effect of adult experience acquired at the time of emergence from pupa on food preference in free-flying flies outside of the laboratory (although other studies failed to find it; see the Introduction). We extend those results in three ways.

First, in previous studies of the effect of experience on food preference in free-flying flies, flies were exposed to the focal odour or flavour upon emergence. These experiments were thus specifically meant to address the “chemical legacy hypothesis” (Corbet 1985), according to which chemical signals encountered by an insect upon emergence may influence its subsequent feeding and oviposition behaviour. In contrast, our flies emerged in standard culture vials and first fed on a standard cornmeal/sugar/yeast medium; they were only exposed to the focal food substrates several (3–10) days after emergence. Thus, our results, in particular experiment 3, indicate that the ability of flies to learn under free-flying semi-natural conditions extends beyond being conditioned to the first food encountered in adult life.

Second, in contrast to those previous field studies, in our study flies were exposed to two substrates, both having an attractive odour but one being highly palatable and the other less so due to presence of quinine. Even though the flies likely spent more time on the more palatable food during the learning phase, the proximity of the two substrates meant that they were exposed to both odours. This excludes simple odour exposure as the cause of the change in subsequent odour preference. Rather, our results are most parsimoniously explained by flies having learnt the association between food odour and its palatability, in analogy to associative learning about food quality observed under laboratory conditions (Mery and Kawecki 2002).

Third, we have shown that memory persists overnight under the free-flying greenhouse setting in the absence of the focal odours, indicating that a consolidated form of memory is involved. Two forms of consolidated memory − long-term memory (LTM) and anaesthesia-resistant memory − have been described in laboratory olfactory classical conditioning in *Drosophila*; they are the only memory forms that persist beyond several hours (Isabel et al. 2004; Davis 2005). In laboratory studies of consolidated memory flies are maintained between conditioning and test under conditions that minimize exposure to odours and other stimuli, which may be particularly favourable to consolidation and retention of memory (Dacher and Smith 2008; Lagasse et al. 2009; Burns et al. 2011). Hoffmann and Turelli (1985) reported apparent retention of memory about food in flies maintained over 24 h under such minimal-stimulus laboratory conditions (vials with no food) and subsequently released and tested in the field. However, this result was inconsistent between experiments and may have been confounded by the degree of food fermentation (Hoffmann 1985); another study reported that the effect of experience disappears in the field within hours (Jaenike 1986). Our results thus provide the strongest support yet for the importance of consolidated memory in *Drosophila* foraging. In our Experiment 2 the flies still showed increased preference for the food they previously experienced as more palatable, even though in the meantime they spent 13–16 h in the greenhouse, in the absence of the focal food odours, but exposed to other odours such as those emanating from potted plants and soil. While part of the overall time spent in the greenhouse was spent roosting on plants as soil (as we observed), the flies are also likely to have actively sought moisture and food, in particular during the natural peaks of activity at dusk and dawn. The stimuli encountered during that time might have interfered with the pre-existing memory (Dacher and Smith 2008; Lagasse et al. 2009). The fact that their memories of food-associated odours from the previous day persisted under these conditions is a strong indication that such memories may also persist in nature. Thus, while we cannot discern whether flies in our assays relied on long-term or anesthesia-resistant memory, our study indicates that consolidated memory may be ecologically relevant even for small, short lived insects.

Overall, our results provide strong support for the notion that fruit flies modify their food choice in a natural context based on previous experience. Learning detected in laboratory conditioning paradigms is thus not merely an artefact of strong stimuli implemented in such assays, small spatial scale and absence of other stimuli. Learning about food and oviposition sites is likely to be important for *D. melanogaster* in nature. Fruit flies feed on decaying fruits, the quality and palatability of which vary in space and time, depending on the stage of decomposition, the microorganisms involved, and the degree of desiccation. Volatile compounds through which flies are attracted to fermenting substrates (Markow and O'Grady 2008) may not reflect the quality of the substrate accurately. If so, it will often be beneficial for flies to be able to adjust their attraction to volatiles based on their local, recent experience. While it has been suggested that fruit flies spend extended periods of time on or near the food sites (Spieth and Heed 1972), they may be driven away temporarily, for example, by the heat of mid-day (Feder 1997), as well as roost away from the feeding sites overnight. If that is the case, retaining learned information over prolonged periods might be useful in relocating temporarily abandoned, but still appropriate food sites, or locating new ones with similar properties. While this remains to be directly demonstrated, our results support the plausibility of such a scenario. They also provide an ecological underpinning for laboratory learning assays involving associations between food and odours and used to study the mechanisms or evolution of learning in this model species (Mery and Kawecki 2002; Chabaud et al. 2006). Finally, fruit flies probably do not possess extraordinary learning abilities compared to other insect groups (Dukas 2008). Hence, our results suggest that the ecological significance of associative learning in insects extends beyond the special cases of social Hymenoptera, parasitoids and butterflies reviewed in the Introduction.
